# Vitamin D Serum Levels in Type 2 Diabetic Patients: A Cross-Sectional Study

**DOI:** 10.7759/cureus.22558

**Published:** 2022-02-24

**Authors:** Hussain A Al Ghadeer, Mohammed S AlRamadan, Mohammed M Al Amer, Meshal J Alshawaf, Fatimah J Alali, Aisha A Bubshait, Maryam A Alramadhan, Zainab Almurayhil, Nasser S Aldandan, Mohammed A AlKhamis, Habeeb A AlHaddad, Abdulatif AlOmair

**Affiliations:** 1 Paediatrics, Maternity and Children Hospital, AlAhsa, SAU; 2 Internal Medicine, King Faisal University, AlAhsa, SAU; 3 Internal Medicine, College of Medicine, King Faisal University, AlAhsa, SAU; 4 Medicine, king Faisal University, AlAhsa, SAU; 5 College of Medicine, King Faisal University, AlAhsa, SAU; 6 Medicine, King Faisal University, AlAhsa, SAU; 7 Internal Medicine, King Faisal University, AlHofuf, SAU

**Keywords:** 25 (oh) vitamin d, cross sectional studies, interna medicine, saudi arabia., diabetes type ii

## Abstract

Background and objective

Type 2 diabetes mellitus (T2DM) is a chronic metabolic disorder characterized by hyperglycemia. It is linked with an increase in morbidity (e.g., blindness, kidney failure, stroke, cardiovascular diseases, limb amputations), premature mortality, high healthcare costs, and is quickly becoming a global epidemic disorder. Several studies have shown that vitamin D supplements reduce insulin resistance in T2DM and improve insulin secretion and sensitivity. In this study, we aimed to determine the prevalence of vitamin D deficiency in T2DM patients in Saudi Arabia.

Methods

This was a retrospective cross-sectional study conducted at the King Faisal University Health Centre in Saudi Arabia. The study used patient data during the period from October 2014 to January 2021. After obtaining approval from the King Faisal University Polyclinic Administration, we collected patient data from the King Faisal University Health Centre. The Ethics and Research Committee at the College of Medicine of King Faisal University granted ethical approval with the approval number (2020-11-82). The relevant patient data were collected, including age, gender, nationality, and blood test findings (vitamin D and HbA1c levels).

Results

A total of 191 T2DM patients participated in this study. The mean age of the patients was 56.1 ± 11.4 years (range: 21-85 years); 107 (56%) patients were females, and 137 (71.7%) were Saudis. There were 134 (70.2%) patients with vitamin D deficiency, 53 (27.7%) with vitamin D insufficiency, and only four (2.1%) with normal vitamin D levels.

Conclusion

Based on our findings, the prevalence of vitamin D deficiency among T2DM patients is highly associated with poor diabetic control.

## Introduction

Type 2 diabetes mellitus (T2DM) is a chronic metabolic disorder characterized by hyperglycemia [1]. It is associated with a significant increase in morbidity (e.g., blindness, kidney failure, stroke, cardiovascular diseases, limb amputations), premature mortality, high healthcare costs, and is rapidly becoming an epidemic disorder of global proportions [2]. The World Health Organization (WHO) predicts that the number of diabetic patients worldwide will exceed 370 million by 2030 [3]. Locally, Saudi Arabia ranks first among Middle Eastern countries in terms of estimated diabetes mellitus cases [4]. Furthermore, various epidemiological studies show that the prevalence of diabetes mellitus is increasing every year, paralleling the increase in life expectancy, as the elderly population is at a higher risk of developing T2DM [5]. Also, multiple studies show that more than 90% of T2DM cases are strongly associated with poor lifestyle, obesity (BMI >30 kg/m^2^), and reduced physical activity [4,5].

Vitamin D is an essential nutrient for humans, which can be obtained both exogenously and endogenously. The primary source of vitamin D is endogenous synthesis by the skin with the help of ultraviolet light [5]. Vitamin D deficiency is indicated if the serum level of 25-hydroxyvitamin D (25(OH)D) falls below the level of 50 nmol/L, while serum concentrations <75 nmol/L are indicative of vitamin D insufficiency [2,4]. According to WHO, more than one billion people are vitamin D-deficient or insufficient worldwide [4,5]. Furthermore, multiple studies estimate that the global prevalence of vitamin D deficiency and insufficiency is between 30 and 87% [6]. Several factors, including genetics, lifestyle-related, environmental, and nutritional, all play a role in the development of vitamin D deficiency and insufficiency [1]. Additionally, an increase in BMI increases the risk of developing vitamin D insufficiency and deficiency because vitamin D is deposited in adipose tissue and becomes biologically inactive [2]. Furthermore, due to the increased need for vitamin D in certain patient groups, these patient groups, such as infants, lactating women, and adolescents, are at a higher risk of developing vitamin D deficiency and insufficiency [2].

According to several studies, vitamin D plays an important role in the prevention of cardiovascular diseases and cancers, inhibition of parathyroid hormone secretion and adaptive immunity, and the promotion of innate immunity [2,7]. Furthermore, vitamin D is primarily involved in glycemic control and the prevention of diabetic complications [1]. Several observational and cross-sectional studies [2,6] have suggested a link between vitamin D deficiency and the development of metabolic syndrome or T2DM and cardiovascular diseases. Vitamin D promotes insulin action by regulating insulin receptor gene expression, which increases insulin sensitivity [2]. Accordingly, vitamin D deficiency is likely to cause insulin resistance, which is the main reason for the development of T2DM [1,8]. Furthermore, insulin secretion is calcium-dependent, and a lack of vitamin D impairs glucose-mediated insulin release [2]. Several studies have shown that vitamin D supplements reduce insulin resistance in T2DM and improve insulin secretion and sensitivity [3,4].

The currently available data in the literature is insufficient to support the link between T2DM and vitamin D deficiency, as large trials are required for the same. In light of this, the goal of this study was to determine the prevalence of vitamin D deficiency in type 2 diabetic patients in Saudi Arabia.

## Materials and methods

Study design

This was a retrospective cross-sectional study conducted at the King Faisal University Health Centre in Saudi Arabia. The study collected patient data from October 2014 to January 2021.

Procedure

Following approval from the King Faisal University Polyclinic Administration, we collected patient data from the King Faisal University Health Centre. The Ethics and Research Committee, College of Medicine, King Faisal University granted the ethical approval (approval number: 2020-11-82). The relevant patient data were gathered, including age, gender, nationality, and blood test results (vitamin D and HbA1c levels).

Inclusion and exclusion criteria

The inclusion criteria were as follows: patients aged more than 18 years old, diagnosed with T2DM >3 months ago, and followed up regarding DM on a regular basis at the King Faisal University Health Centre.

The exclusion criteria were as follows: patients aged <18 years, those who were recently diagnosed with T2DM, patients diagnosed with DM type I, patients presenting with no comorbid diseases related to abnormal lipids profile, e.g., endocrine, renal, or hepatic disorders.

Research population

A total of 191 T2DM patients who were one year or older were included in our study.

Materials

The cut-off values for the HbA1c level were from 4 to 5.9%. The cut-off levels of vitamin D level were divided into the following ranges - normal: 50%-70 ng/ml, insufficient: 30-49 ng/ml, and deficient: <30 ng/ml.

Data analysis

Two-tailed tests were used for all statistical analyses. A p-value of less than 0.05 was considered statistically significant. All variables, including patients' demographic data, HbA1c, and vitamin D level, were subjected to descriptive analysis based on the frequency and percentage distribution. The lipid profiles were shown as means with standard deviations. The Pearson X^2^ test and exact probability test for small frequency distributions were used to test the relationship between vitamin D levels and type 2 diabetic patients' demographic data and HbA1c levels. Next, correlation analysis was used to determine the relationship between vitamin D levels and the patients' lipid profiles and HbA1c levels, using SPSS Statistics version 22 (IBM, Armonk, NY).

## Results

A total of 191 T2DM were included in the study. The mean age of the patients was 56.1 ± 11.4 years (range: 21-85 years). The cohort comprised 107 (56%) females and 137 (71.7%) Saudis (Table 1).

**Table 1:** Demographic data of the patients

Variables	N	%
Age group, years		
<40	17	8.9%
40-59	96	50.3%
60+	78	40.8%
Gender		
Male	84	44.0%
Female	107	56.0%
Nationality		
Saudi	137	71.7%
Non-Saudi	54	28.3%

**Figure 1:**
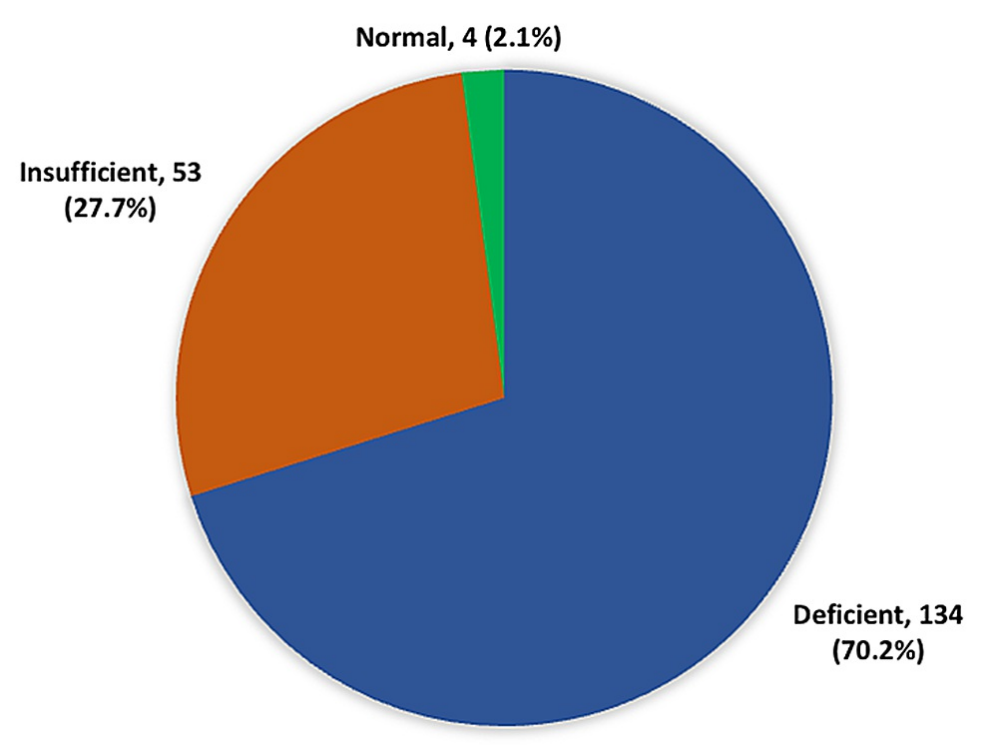
Vitamin D deficiency among type 2 diabetic patients

A total of 134 (70.2%) patients were vitamin D-deficient, 53 (27.7%) had insufficient vitamin D levels, and only four (2.1%) had normal vitamin D levels (Figure 1).

Of note, 178 (93.2%) presented with HbA1c levels >5.9%, and 13 (6.8%) presented with HbA1c levels <5.9% (Figure 3).

**Figure 2:**
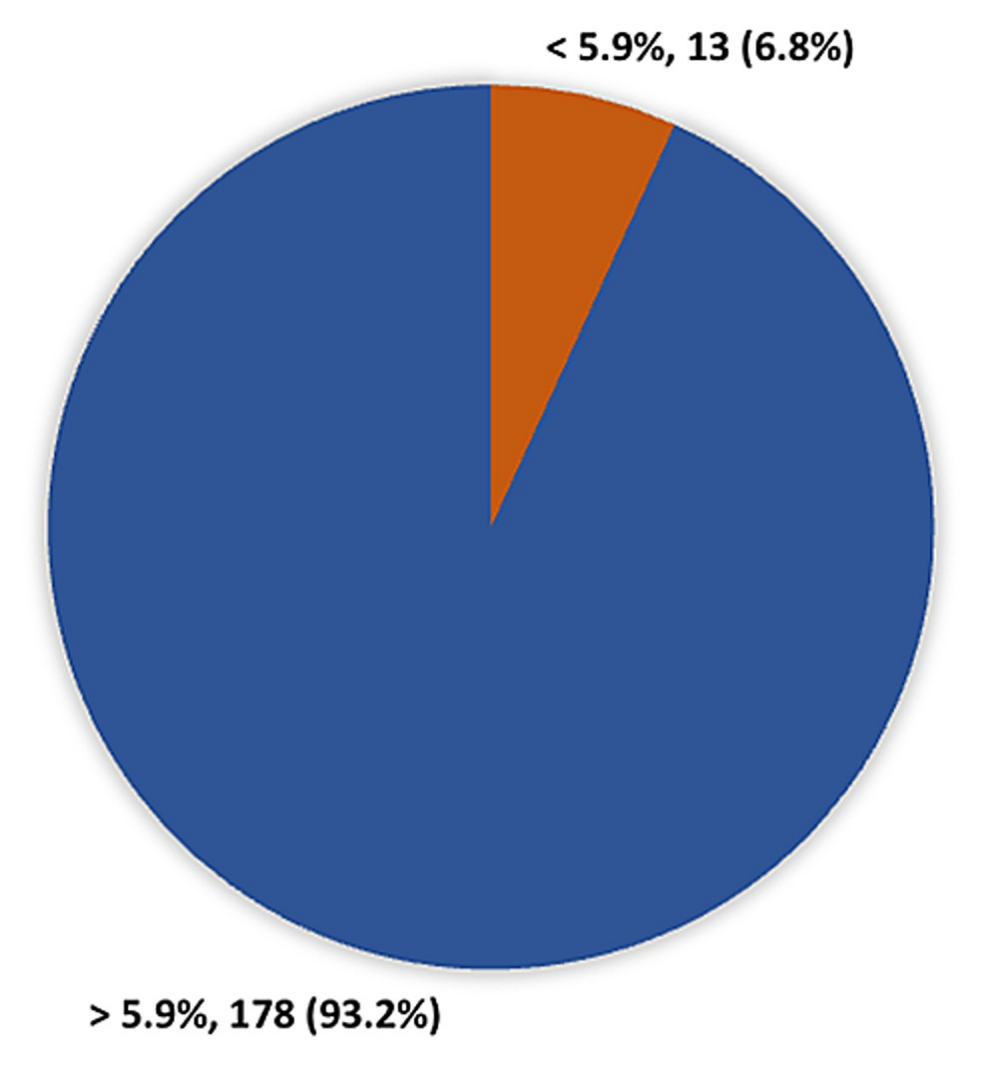
HbA1c levels among type 2 diabetic patients

Vitamin D deficiency was detected among 76.5% of patients aged less than 40 years, compared to 60.3% of those aged 60 years or more; however, this difference had no statistical significance (p=0.162). Also, 71.4% of male patients had presented with vitamin D deficiency versus 69.2% of females, again with no significant difference (p=0.894). On the other hand, 71.9% of diabetic patients with HbA1c above 5.9% had presented with vitamin D deficiency in comparison to 46.2% of those with HbA1c levels less than 5.9%, and this difference was statistically significant (p=0.048) (Table 2).

**Table 2:** Distribution of vitamin D deficiency by patients’ personal data and HbA1c levels *Pearson X^2^ test. ^$^Exact probability test. **P<0.05 (significant)

Factors	Vitamin D level						P-value*
	Deficient		Insufficient		Normal		
	N	%	N	%	N	%	
Age group, years							0.162^$^
<40	13	76.5%	4	23.5%	0	0.0%	
40-59	74	77.1%	20	20.8%	2	2.1%	
60+	47	60.3%	29	37.2%	2	2.6%	
Gender							0.894
Male	60	71.4%	22	26.2%	2	2.4%	
Female	74	69.2%	31	29.0%	2	1.9%	
HbA1c level							0.048**^$^
<5.9%	6	46.2%	7	53.8%	0	0.0%	
>5.9%	128	71.9%	46	25.8%	4	2.2%	

## Discussion

Many diseases, including T2DM, are linked with vitamin D deficiency [9]. Whether this relationship is causal or confounding is still a matter of debate. The active metabolite 1α,25-dihydroxy vitamin D3 (1,25(OH)2D3) affects pancreatic β cells and insulin secretion, and besides other factors, it may influence insulin sensitivity [10]. The vitamin D receptor (VDR) is present in many organs, which explains the fact that vitamin D metabolites may show numerous extra-skeletal effects [11]. A link exists between vitamin D deficiency and insulin resistance due to inflammation, as vitamin D deficiency is accompanied by higher inflammatory markers [12]. Nevertheless, a Mendelian randomization study on vitamin D and C-reactive protein failed to indicate a causal relationship [13].

The current study sought to assess vitamin D deficiency among type 2 diabetic patients in King Faisal University Health Centre in Saudi Arabia. The results showed that about one-fifth of the patients presented with vitamin D insufficiency, and slightly less than three-quarters presented with vitamin D deficiency (70.2%), meaning nearly all cases presented with either vitamin D deficiency or insufficiency. These findings are consistent with those of Alhumaidi et al., who found that 98.5% of type 2 diabetic patients presented with 25-OH vitamin D deficiency [3]. The mean serum 25-OH vitamin D levels in the diabetic group were 15.7 + 7.5 ng/mL. Al-Zahrani et al. reported that 98.4% of type 2 diabetic patients presented with vitamin D deficiency [14]. Nearly 75% of female diabetic patients presented with vitamin D deficiency compared to less than half of male diabetic patients (46.9%), but 50.8% and 25.6% presented with vitamin D insufficiency. Bajaj et al. found that vitamin D deficiency (<20 ng/ml) was detected in 59.5% of type 2 diabetic patients, and their mean vitamin D level was 19.046 ± 6.614 ng/ml [15].

In addition, many other studies have found a link between vitamin D deficiency and T2DM. The Mini-Finland Health Survey evaluated T2DM subjects and found a significant negative association between serum vitamin D levels and the risk of T2DM [16]. Vitamin D deficiency was found in 91.1% of diabetic subjects in India, according to Daga et al. Approximately 60% of diabetic cases presented with high vitamin D deficiency, compared to 8.3% of controls [17]. In the current study, patients with poor diabetic control presented with the highest level of vitamin D deficiency, with nearly three-quarters of those with HbA1c greater than 5.9% presenting with vitamin D deficiency compared to less than half of those with HbA1c less than 5.9%. This can be explained by the reported link between vitamin D deficiency and insulin resistance among diabetic patients. Many studies have assessed the link between vitamin D and the physiological function of the pancreatic β cell as β cells express VDRs, and 1a-hydroxylase is assessed in pancreatic tissue, equivalent with the expression of insulin [18]. Insulin secretion depends on calcium level, and it has been noted that vitamin D deficiency prevents glucose-facilitated insulin secretion [18,10]. Supplementation with vitamin D enhances insulin secretion based on oral glucose levels, with an increase in serum calcium and a decrease in free fatty acids [17].

## Conclusions

The current study found that nearly all cases of T2DM presented with either vitamin D deficiency or insufficiency, with nearly one out of three patients presenting with vitamin D deficiency. Furthermore, vitamin D deficiency is linked to poor diabetic control due to a reported association with increased insulin resistance or a defect in insulin release.
